# Biomechanical analysis of little penguins’ underwater locomotion from the free-ranging dive data

**DOI:** 10.1242/bio.060244

**Published:** 2024-05-23

**Authors:** Mahadi Hasan Masud, Peter Dabnichki

**Affiliations:** ^1^ Department of Mechanical Engineering, Rajshahi University of Engineering and Technology, Rajshahi 6204, Bangladesh; ^2^ School of Engineering, RMIT University, Bundoora Campus, Melbourne, VIC, Australia 3083

**Keywords:** Little penguin, Propulsion, Hydrodynamic forces, Energy cost, Strouhal number

## Abstract

Penguins are proficient swimmers, and their survival depends on their ability to catch prey. The diving behaviour of these fascinating birds should then minimize the associated energy cost. For the first time, the energy cost of penguin dives is computed from the free-ranging dive data, on the basis of an existing biomechanical model. Time-resolved acceleration and depth data collected for 300 dives of little penguins (*Eudyptula minor*) are specifically employed to compute the bird dive angles and swimming speeds, which are needed for the energy estimate. We find that the numerically obtained energy cost by using the free-ranging dive data is not far from the minimum cost predicted by the model. The outcome, therefore, supports the physical soundness of the chosen model; however, it also suggests that, for closer agreement, one should consider previously neglected effects, such as those due to water currents and those associated with motion unsteadiness. Additionally, from the free-ranging dive data, we calculate hydrodynamic forces and non-dimensional indicators of propulsion performance – Strouhal and Reynolds numbers. The obtained values further confirm that little penguins employ efficient propulsion mechanisms, in agreement with previous investigations.

## Teaser

**Summary:** The analysis of free-ranging dive data for little penguins shows that, as expected, they employ efficient propulsion mechanisms and dive in a way that minimises cost of transport.

## INTRODUCTION

The world is currently experiencing severe variations of weather patterns in general and rising temperatures. These catastrophic changes alter the polar areas and the oceans surrounding them. Many species are affected as their fortunes are inextricably linked to the health of the oceans, whose resources are further depleted by destructive commercial activities such as non-sustainable fishing and krill harvesting for food supplements (Ropert-Coudert et al., 2019). The survival rate of penguins depends on their ability to forage in the ocean, an activity that requires energy to find and gather food (Careau et al., 2003). The penguins’ ability to catch prey is a direct consequence of their swimming ability, i.e., one expects that the survival of these seabirds depends on their proficiency as swimmers. The statement is to some extent proven by the relatively large number of studies focusing on how penguins move underwater ‒ Harada et al. (2021), Masud et al. (2022) and Masud et al. (2023) are recent examples. Anomaly in the energy expenditure pattern of penguins can potentially signal early signs of disturbance in their ecosystem. Hence, quantitative energy study can be used as an essential fitness indicator for penguins, which is directly correlated to their efficient propulsion. To understand the energy requirements, one needs to gain an in-depth understanding of the propulsion energy and power requirements; the first step of which is to devise an accurate estimate of the occurring hydrodynamic forces. However, energy expenditure associated to penguins’ underwater locomotion are not investigated sufficiently in terms of the mechanical work necessary for the dives. Although, Sato et al. (2010) predicated and mentioned the values of energy expenditure for penguin locomotion, but those are not estimated using free ranging dive data. Harada et al. (2021) and Masud et al. (2022) also demonstrate that quantitative information is still lacking, particularly for free-ranging birds.

Specifically, corresponding theoretical models have been developed over the years ‒ see, for example, Lovvorn et al. (2001) and Sato et al. (2010) ‒ but they are in general based on assumptions that should be tested, for example, that water temperature and weather do not affect the mechanical work significantly (see Materials and Methods, where all the assumptions are mentioned in detail). The current work presents such a test. Although previously Culik et al. (1994) reported the cost of transport (CoT) for Adélie (*Pygoscelis adeliae*), Chinstrap (*P. antarctica*) and Gentoo (*P. papua*) penguins based on respirometry, but they did so only for swimming in a confined canal, and also, they did not consider the mechanical cost of transport (Culik et al., 1994).

For the first time, free-ranging dive data are employed to estimate the forces acting on little penguins and the associated energy cost is computed on the basis of the biomechanical model developed by Sato et al. (2010). From time-resolved acceleration and depth data already used in a previous study by MacIntosh et al. (2013), swimming speed and pitch angle are calculated for 300 dives of little penguins (MacIntosh et al., 2013). The latter quantities are then used to estimate the buoyancy and drag forces, contributing to the mechanical energy cost, as discussed by Sato et al. (2010).

In short, we find that the energy cost computed from the present free-ranging data is of the same order of the minimum expenditure predicted by the chosen biomechanical description by Sato et al. (2010). The result, therefore, supports the physical soundness of the latter description, and we also suggest possible directions for model improvement, especially those focusing on unsteady effects. Moreover, by implementing the same biomechanical model, we have estimated the hydrodynamic forces acting on the free-ranging little penguin. Finally, following the study of Masud et al. (2022), we compute the non-dimensional indicators of propulsion performance, confirming that, as expected, penguins employ efficient propulsion mechanisms (Masud et al., 2022).

## RESULTS

### Diving characteristics

The present analysis is based on free-ranging dive data recorded in Australia approximately ten years ago. Specifically, the accelerations of free-ranging little penguins were collected along three directions and at the same time, the bird depth was also recorded (see Materials and Methods for details).

If one plots the current depth data as a function of time, dives can be easily identified and, for our analysis, we chose some exemplary cases (see Table 1 for relevant properties). Dives are here categorized depending on the dive depth achieved by the bird in the chosen situation, and we computed the bird speed from the corresponding depth and acceleration data, following Watanuki et al. (2003); see also Materials and Methods.

**Table 1.**
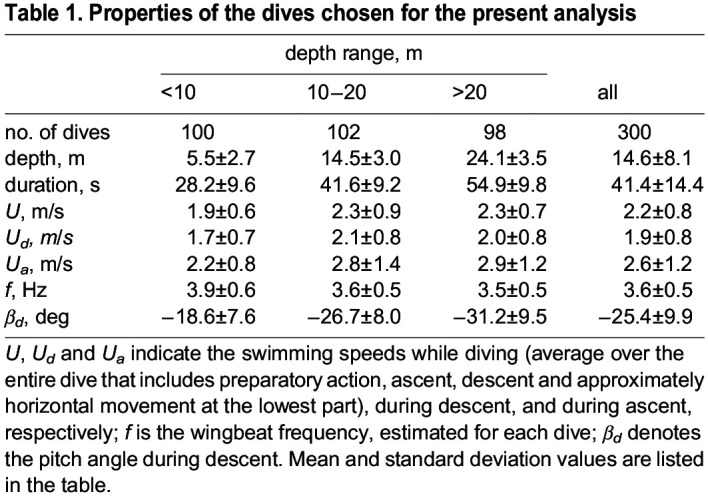
Properties of the dives chosen for the present analysis

From Table 1*,* it is evident that, in the range of investigated parameters, the swimming speed is always greater during ascent, when the bird returns to the sea surface, than during descent. This is consistent with the fact that the buoyancy force acts in the direction opposite of gravity. Additionally, the buoyancy force is inversely proportional to depth, as discussed, for example, by Sato et al. (2010). Consistently, the difference between the values of swimming speeds during ascent and descent, i.e., *U*_*a*_−*U*_*d*_, shows the tendency to become higher with increasing depth, especially for the deepest dives.

In Fig. 3, the relative acceleration and current depth of a typical dive are plotted as a function of time (the relative acceleration is defined as the difference between the acceleration magnitude and the absolute value of the acceleration due to gravity, see Materials and Methods for the formula). Note that the same acceleration range for free-ranging little penguins was reported in Watanuki et al. (2006). Additionally, one clearly sees that, at the end of the ascent, acceleration changes are substantially lower than during any other period of the dive. This likely indicates that stroke cessation occurred, i.e.*,* the bird took advantage of the buoyancy force while returning to the sea surface and significantly decreased the movements of its flippers. The phenomenon was observed in the majority of the chosen dives – almost 80% – and more often for the deepest dives, when it also seemed to start earlier, at a relatively deeper location. The outcome is in qualitative agreement with previous results by Sato et al. (2002); however*,* the large scatter associated with the identification of stroke cessation in the current data does not allow*,* at present*,* more quantitative remarks.

Another relevant feature of the captured acceleration data can be seen in Fig. 4, which displays the same data plotted in Fig. 3 for two chosen time intervals, at the dive beginning (left panels) and at the dive bottom (right panels). It is evident that the *wingbeat* frequency – associated to the bird propulsive frequency, for example, in Sato et al. (2002) – is larger at the start of the dive descent than at the dive bottom. The phenomenon was specifically observed for flat-bottomed dives, occurring mainly when the bird is looking around for prey. For dives having different trends, e.g.*,* for bounce dives, regions of high-frequency acceleration were also found at the bottom, while the bird was most likely already hunting and not just looking for prey; see, for example, Wilson et al. (1996) for a discussion on penguins’ dive shapes (Wilson et al., 1996).

Nevertheless, the frequency increase observed for some dives in the proximity of the sea surface can also be related to the fact that, at the dive beginning, the penguin needs to overcome the buoyancy force, which decreases with increasing depth. Similarly, one can note in Table 1 that the wingbeat frequency tends to be smaller for deeper dives. Additionally, the obtained frequency values are very similar to those reported by Watanuki et al. (2006) for free-ranging little penguins (Watanuki et al., 2006).

From the acceleration data*,* we also computed the dive (or pitch) angle, indicating the bird swimming direction (see Materials and Methods for the formula). This angle is set negative when the penguin is descending, and it is null when the bird swims horizontally. It is positive when the penguin returns to the sea surface, while ascending.

In Fig. 5, the pitch angle is plotted as a function of time for several dives. It is apparent that its values can be relatively large, up to about 70° in magnitude. Additionally, closer inspection of the data reveals that shallower dives tend to be characterized by lower dive angles than deeper dives (see Table 1 for relevant values). The outcome is consistent with theoretical results reported by Sato et al. (2010), obtained using the biomechanical model outlined above, which was developed to assess the energy cost during descent (Sato et al., 2010). Specifically, it was found that this cost is minimum for vertical descent, corresponding to a dive angle of −90°, and that, for a −70° pitch angle, the energy cost is only slightly larger. In other words, one could say that steeper dives are more likely to occur when the bird is actively hunting and rather than scanning for prey.

As already noted, we computed the swimming speed from the acceleration and depth data, following Watanuki et al. (2003), the main reason being that, in the model devised by Sato et al. (2010), the energy cost during descent depends on the dive depth, swimming speed and pitch angle. Additionally, as discussed, for example, by Masud et al. (2022), the drag force acting on an object moving in a fluid is often set proportional to the square of the object speed and, as detailed by Sato et al. (2010), this force directly contributes to the energy cost that can be associated to the dive descent, similarly to the buoyancy force, which, as already noted, is inversely proportional to the depth (see Materials and Methods for details on the chosen force and energy formulas, taken mostly from by Sato et al. (2010).

### Hydrodynamic force

In Fig. 6, the drag and buoyancy forces are plotted as a function of time for an exemplary dive, together with the corresponding speed and depth. As already mentioned, the buoyancy force is smallest at the dive bottom. Additionally, the drag force is directly proportional to the square of the swimming speed, i.e., the speed values listed in Table 1 are directly related to the square root of the average drag force acting on the bird.

### Cost of transport

We have now gathered all the information needed to estimate the energy cost *E*_*C*_ associated to the dive descent, on the basis of the above-mentioned biomechanical model, developed by Sato et al. (2010). Specifically, we employed mean values of dive depth, swimming speed, and pitch angle estimated from the free-ranging dive data to calculate for the first time the average energy cost during descent for free-ranging little penguins. We, therefore, check here the soundness of the proposed biomechanical model by Sato et al. (2010).

First, in Table 2, we list the computed energy cost values, together with the average values of the depth of the free-ranging little penguin dive, swimming speed, and pitch angle during descent – see also Table 1. It is apparent that *E*_*C*_ is larger for deeper (and steeper) dives, while the opposite is true for the cost of transport *CoT*, which is inversely proportional to the depth.

**Table 2.**
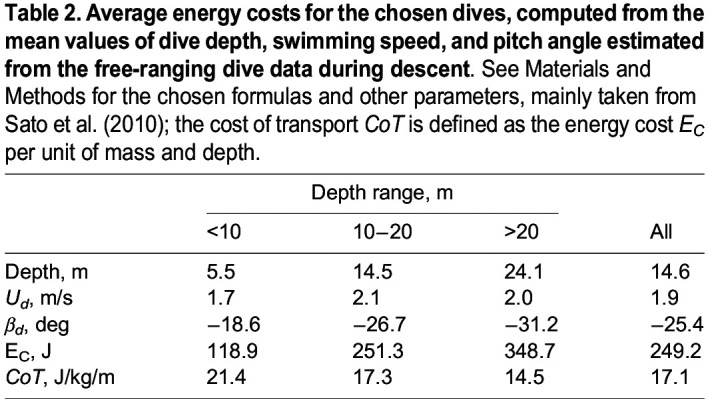
**Average energy costs for the chosen dives, computed from the mean values of dive depth, swimming speed, and pitch angle estimated from the free-ranging dive data during descent**. See Materials and Methods for the chosen formulas and other parameters, mainly taken from Sato et al. (2010); the cost of transport *CoT* is defined as the energy cost *E_C_* per unit of mass and depth.

The comparison with the model, which can be regarded as the main result of this work, is displayed in Fig. 7. Specifically, at a depth equal to the actually obtained average depth for all dives performed by the little penguin, i.e., 14.6 m, we computed the energy cost as a function of swimming speed and dive angle during descent. The result is plotted as an energy map. The white point on this map indicates the actually obtained *E*_*C*_ value for all dives, corresponding to the data listed in the last column of Table 2 – note in passing that the map appearance does not change significantly with depth but the *E*_*C*_ magnitude does increase with depth.

As mentioned above, the model predicts that the energy cost is minimum for vertical descent, corresponding to a dive angle of −90°, implicitly assuming that the bird already knows where to look for prey, i.e., the model does not consider exploratory dives. Similarly, we argue here that the swimming speed associated to the energy cost minimum is different from the one they actually experienced. Additionally, the model does not account for water currents, which may influence the penguins’ swimming behaviour, in analogy to what was reported for salmons by Johansson et al. (2014). On the other hand, the calculated *E*_*C*_ value from the free-ranging penguin dive data is about two and a half times larger than the minimum one, i.e., it is still relatively low, in comparison with other cases reported in Fig. 7.

## DISCUSSION

In order to better appreciate the comparison with the real free-ranging penguins dive data displayed in Fig. 7, here, we discuss in more detail the biomechanical model devised by Sato et al. (2010). Specifically, the formula employed for estimating the energy cost, which is reported in Materials and Methods, is made of three terms. The first two terms are associated to the mechanical energy needed to reach a given depth – they are obtained from the integration of buoyancy and drag forces. The first term slowly increases with depth, because of the logarithmic dependence of the buoyancy force integral. The second term is directly proportional to the depth, i.e., it increases with depth faster than the first term, but it also depends on the square of the swimming speed, and it is inversely proportional to the sine of the dive angle during descent. The third term represents the basal metabolic energy, needed to keep the bird alive at rest. It is directly proportional to the dive depth and inversely proportional to the vertical speed during descent, equal to *U*_*d*_ sin *β*_*d*_.

It then follows that, at given depth and pitch angle, the increase of the second term with swimming speed is slower than the corresponding decrease of the third term, i.e. the speed associated with the minimum energy is smaller than that obtained by equating second and third terms. Additionally, at a given depth, similar trends are observed at increasing pitch amplitude but with smaller energy values. Indeed, this is apparent from the free-ranging dive data listed in Tables 1 and 2. Consequently, the present free-ranging dive data broadly support the physical soundness of the assumptions at the basis of the biomechanical model developed by Sato et al. (2010).

However, the value of energy cost obtained from the free-ranging dive data, also plotted in Fig. 7, is significantly larger than the minimum one, although it is of the same order of magnitude. The discrepancy is likely due to the fact that the model does not account for the foraging behaviour of penguins, i.e., it does not consider exploratory dives, as already noted. It would then be useful to compute the energy cost only for the dives associated to the portion of dives involving pursuit and prey capture but, at present, we cannot identify these dives in the analysed free-ranging dive data.

Similarly, the fact that the observed swimming speed is about 0.5 m/s larger than the one corresponding to the energy cost minimum could be due to the chosen pool of dives. Additionally, the swimming speed and direction of free-ranging penguins could be influenced by ocean currents, which can be rather fast, up to 1 m/s, as reported, for example, by Shirah et al. (2012). We then expect that closer agreement with the model theoretical findings could be observed after removing water current effects from the data, or for dives in still water, but, at present, we cannot identify these dives in the analysed free-ranging dive data.

At this point, it is useful to remark that the biomechanical model developed by Sato et al. (2010) assumes that the dives occur at a constant speed, i.e., the formula chosen for the drag force is in general valid only for steady flows. On the other hand, the acceleration signal obtained from the free ranging dive data ‒ e.g., that plotted in Fig. 3 ‒ clearly indicates that penguins’ speed is not constant for large portions of the dives. Additionally, these time-varying accelerations can be neatly associated to the bird propulsive strokes, as discussed, for example, by Sato et al. (2002). The discrepancy between the model and free-ranging dive data, observed in Fig. 7, could then be also related to the fact that unsteady effects are neglected in the considered biomechanical description, although, at present, we have not quantified their specific contribution to the overall energy cost.

In this regard**,** the wingbeat frequency could play an important role because this quantity depends on the dive depth, as shown, for example, in Fig. 3 ‒ see also Table 1. If one considers that also the buoyancy force depends on the dive depth, it then follows that the two phenomena are most likely related, as already noted. Indeed, at the beginning of the dive, the penguin needs to overcome the buoyancy force, and therefore the generated thrust force should be larger than at the dive end because the buoyancy force acts in the direction opposite to gravity ‒ the argument implicitly assumes that the thrust force increases with frequency, at least in the range of parameters here considered, in agreement with previous studies, e.g., Schouveiler et al. (2005).

Starting from the wingbeat frequency obtained from the heaving acceleration, one can also discuss the overall propulsive performance of the animal, following, for example, Gazzola et al. (2014); that is, non-dimensional indicators of propulsive performance can be computed, and in Table 3 we list relevant values of Strouhal and Reynolds numbers, which were calculated as in Masud et al. (2022) (see Materials and Methods for the formulas). The Strouhal number can be seen as an indicator of the motion unsteadiness. It is directly proportional to the propulsive frequency and amplitude, and it is inversely proportional to the swimming speed. The latter is in general perpendicular to the direction of the propulsive action, and it is directly proportional to the Reynolds number, which is also inversely proportional to the fluid kinematic viscosity, i.e., high ***Re*** means in general high speed and/or low fluid resistance to motion, for a given body geometry.

**Table 3.**
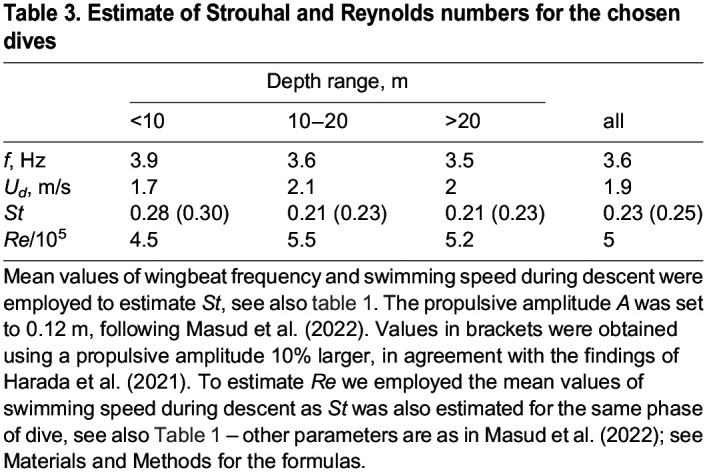
Estimate of Strouhal and Reynolds numbers for the chosen dives

Specifically, it was reported by Taylor et al. (2003) that, for flying and swimming animals, efficient propulsion is more likely to occur within a relatively narrow range of Strouhal numbers, 0.2<St<0.4. Masud et al. (2022) show that this is also the case for penguins, at least at sufficiently large Reynolds numbers, larger than approximately 10^4^ – see Taylor et al. (2003); Eloy (2012); Gazzola et al. (2014) for details on related scaling laws. The value of ***St*** reported by Masud et al. (2022) for little penguins, 0.23, is equal to that in the last column of Table 3 (Masud et al., 2022). Additionally, the propulsive amplitude A was set here to 0.12 m, as by Masud et al. (2022), i.e. it was assumed that A is equal to the bird wingspan (Masud et al., 2022). Therefore, in order to get larger Strouhal numbers using the experimentally obtained U and f values, one should employ larger amplitudes. The latter could be justified on the basis of the findings of Harada et al. (2021), where the propulsive amplitude of gentoo penguins swimming horizontally in a water tank was experimentally found to be approximately 10% larger than the corresponding wingspan.

In other words, the present results obtained from the free-ranging dive data seem to support the notion that propulsive amplitudes for penguins could be larger than their wingspan because, if this is the case, one would obtain Strouhal numbers within the efficient propulsion interval ‒ this is demonstrated in Table 3, where the *St* values in brackets were computed following such an assumption. On the other hand, as discussed, for example, by Masud et al. (2022), Strouhal and Reynolds numbers should only be regarded as qualitative measures of propulsion performance, considering especially that in general propulsive amplitudes change along the wingspan.

Nevertheless, from Table 3, it is apparent that the computed Strouhal numbers show the tendency to decrease with dive depth. The outcome could be related to the above-mentioned need to generate large propulsive forces at the dive beginning, when the buoyancy force is large too ‒ once more, we assume here that larger *St* means larger thrust, at least within the efficient propulsion interval, in agreement with previous studies, e.g., Floryan et al. (2018).

Now, before concluding, it is worth mentioning that the present results are based on dives of free-ranging little penguins; that is, the use of non-dimensional numbers and scaling arguments supports the validity of these results also for other species, at least within certain boundaries, to be determined in due course. For example, one expects that also for larger penguins the propulsive frequency for deep dives will be on average smaller than that for shallow dives but, at the same time, one should take into account that the propulsive frequency for larger species is in absolute value smaller than that associated to smaller ones, considering especially that *St* is of the same order across several penguin species, as shown by Masud et al. (2022).

### Conclusion

The present work belongs to the active and challenging field of study focusing on the swimming behaviour of penguins. Indeed, understanding how penguins move underwater is not only important in its own right, e.g., to appreciate the survival strategy of these fascinating seabirds, but it can also provide critical biomimicry design insights for future research. To achieve this end, one needs both theoretical models and free-ranging dive data, and here we specifically tested the validity of the biomechanical model developed by Sato et al. (2010) using first-hand free-ranging dive data of little penguins.

From acceleration and depth data we calculated swimming speed and dive angle, needed to estimate the energy cost associated to the dive descent. We found that the energy cost computed from the free-ranging dive data is larger than the minimum cost predicted by the model but of the same order of magnitude. The outcome, therefore, broadly supports the physical soundness of the chosen biomechanical model but, at the same time, it indicates that neglected effects should be considered for understanding the penguin swimming behaviour in detail. Specifically, more attention should be given in future studies to unsteady effects, e.g. to the fact that the propulsive frequency depends on the dive depth in a measurable way, i.e. on the time-varying forces that the bird must overcome to reach a certain depth.

We also computed the Strouhal and Reynolds numbers associated to the little penguin dives, from the wingbeat frequency and swimming speed obtained from the free-ranging dive data. We found that, as expected, the obtained values are within the efficient propulsion interval. Additionally, our results suggest that penguins’ propulsive amplitudes could be on average larger than their wingspan, supporting therefore the important role of wing flexibility on propulsion performance, recently discussed by Harada et al. (2021) for gentoo penguins.

In summary, the present work gives a significant contribution to the current understanding of penguins’ propulsion mechanisms, mainly because previous theoretical assumptions have been here confirmed using first-hand free-ranging dive data. The obtained results also indicates that future studies should focus on quantifying the importance of unsteady effects for the energy cost associated to underwater locomotion.

## MATERIALS AND METHODS

### Data collection

Data for this study were gathered in collaboration with the Phillip Island Nature Parks research team in Victoria, Australia, who conducted their fieldwork at Phillip Island's Penguin Parade colony (38031′S, 145009′E), Australia. As a result, location and data-gathering techniques are identical to their strategy (for additional details, see MacIntosh et al., (2013). The following is a short description of a pertinent aspect of the data collection procedure.

**Fig. 1.**
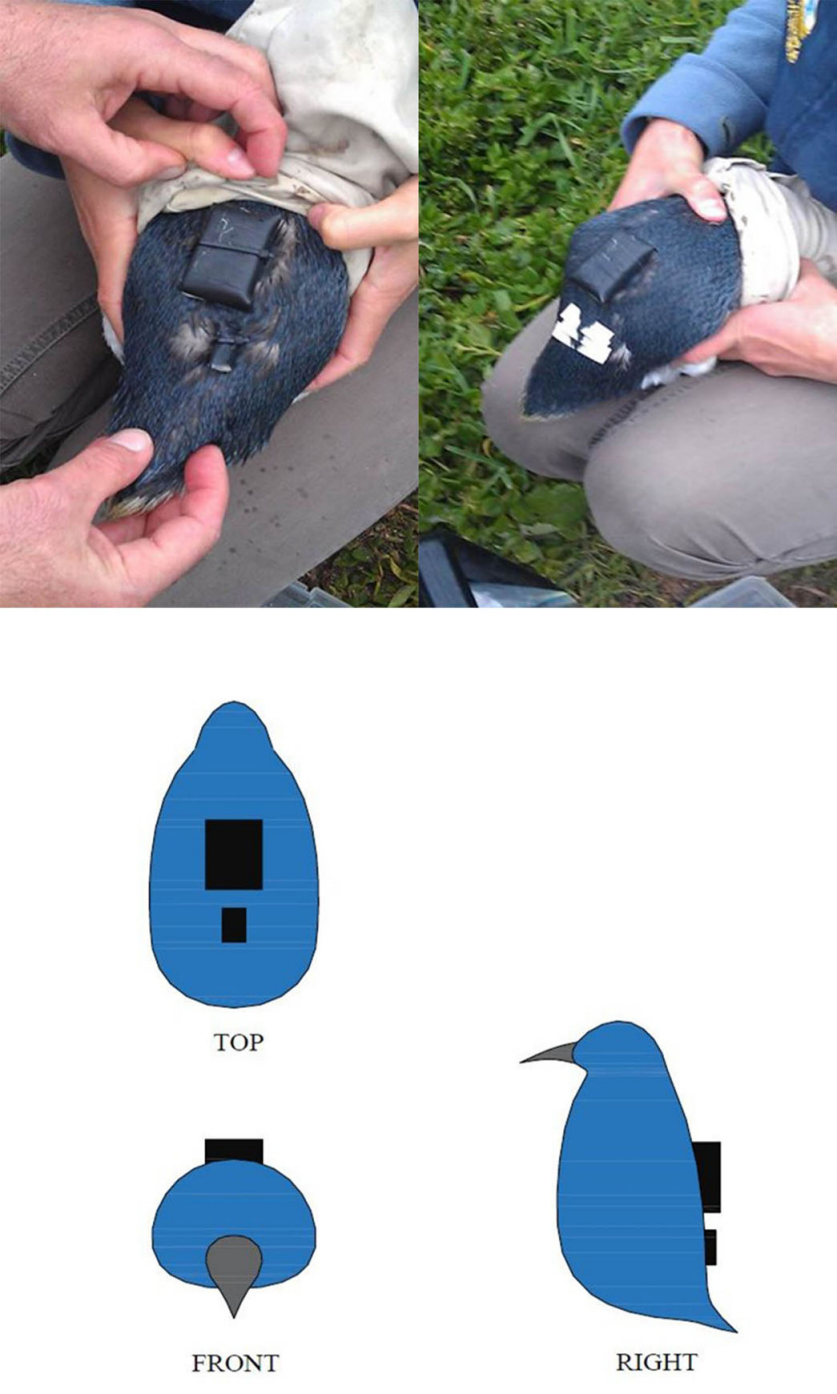
Attachment of data loggers.

The research was carried out with free-living little penguins at the Penguin Parade, Phillip Island (38031′S, 145009′E), Victoria, Australia, during the guard stage of the 2010 breeding season (October 26 – November 26). The diving data collected represent full-day foraging trips of little penguins guarding 1- to 2-week-old chicks. With the goal of reducing drag and facilitating quick deployment and fast removal upon recapture, data loggers were affixed along the median line of the lower back feathers of penguins using waterproof Tesa^®^ tape (Beiersdorf AG, Hamburg, Germany) (see Fig. 1 for details) (Bannasch et al., 1994; Wdson et al., 1997). This allowed it to record any acceleration along the bird's front–rear (surging acceleration) and back–belly (heaving acceleration) axes (Ropert-Coudert et al., 2006). Data loggers (ORI400-D3GT 16-bit resolution accelerometer) were used to collect the acceleration and depth data at sampling frequencies of 50 Hz and 1 Hz, respectively. The same article MacIntosh et al. (2013) contains all the technical information on the data logger (ORI400-D3GT 16-bit resolution accelerometer) (MacIntosh et al., 2013). Moreover, it is essential to mention that both the Department of Sustainability and Environment of Victoria, Australia and the Phillip Island Animal Experimentation Ethics Committee (2.2010) approved the fieldwork (number 10006148).

### Data analysis

The raw acceleration data were collected along the front–rear (surging acceleration) and back–belly (heaving acceleration) axes of the bird. The acceleration data was also collected on the third axis, which is orthogonal to the heaving and surging directions. The current record lasts for 14 h 56 min and includes data obtained from 28 free-ranging penguins. Before analysing the collected data, the raw acceleration data were filtered using a second-order Butterworth low pass filter (cut-off frequency: 0.5) in MATLAB R2019a, and then a *movmean* (moving average over 2 points) filter was also applied to smooth out short-term fluctuations of the acceleration data and highlight longer-term trends.

**Fig. 2.**
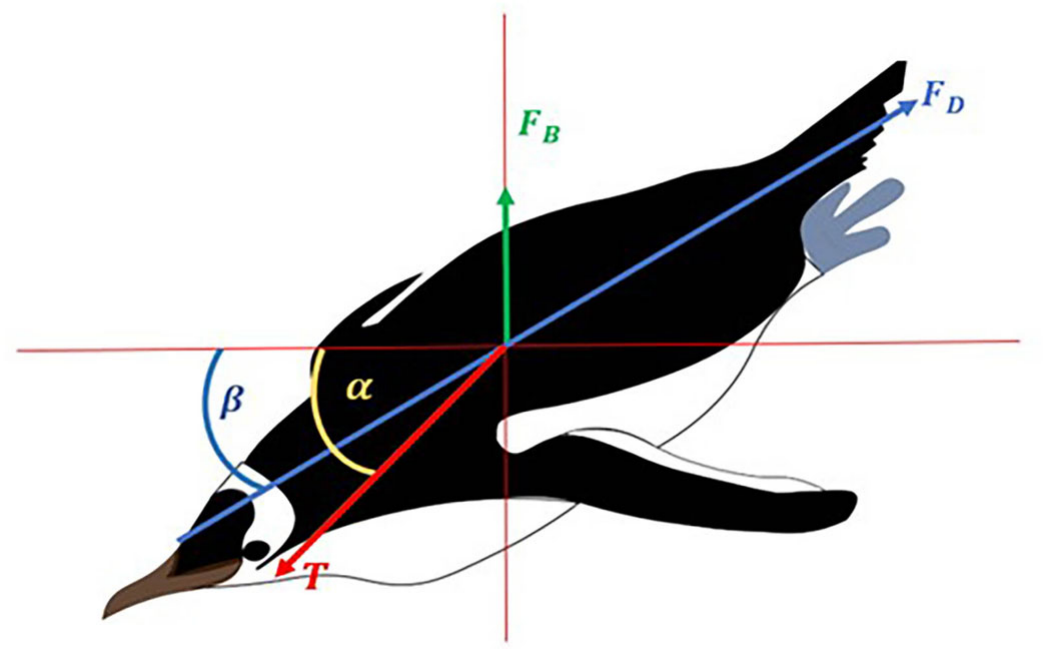
**Schematic diagram of a descending penguin showing all the forces acting on it (here, *β*=pitch angle (deg), *α*=thrust angle (deg), *F_D_*=drag force (N), *F_B_*=Buoyancy force (N), and *T*=thrust force(N)) (Adapted from** **Del Caño et al., 2021****)**.

Fig. 2 shows a schematic diagram of a descending little penguin, which swims against the positive buoyancy, and the downward component of the thrust acts against it (Adapted from Del Caño et al., 2021).

**Fig. 3.**
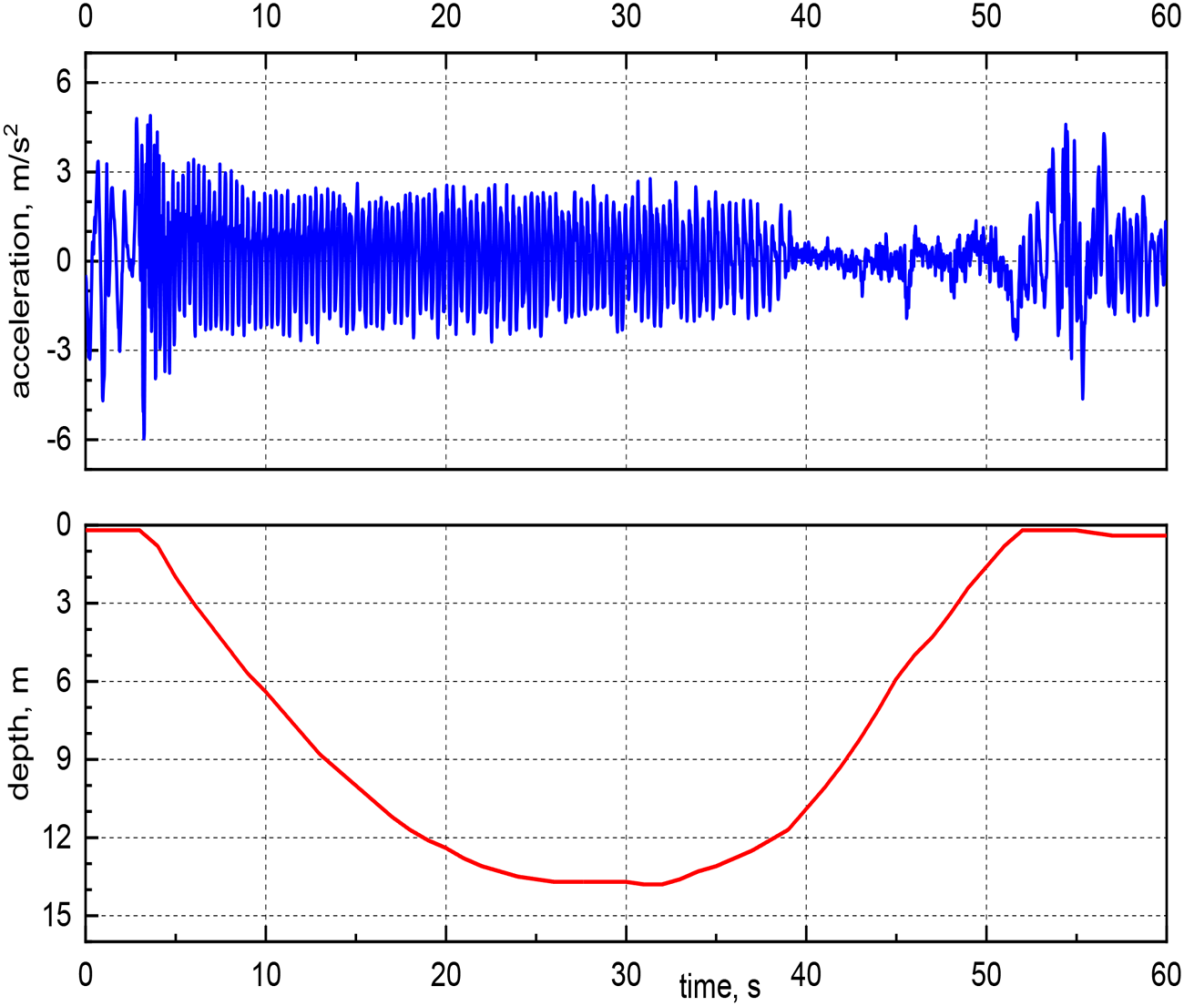
Relative acceleration and current depth as a function of time for a typical dive.

**Fig. 4.**
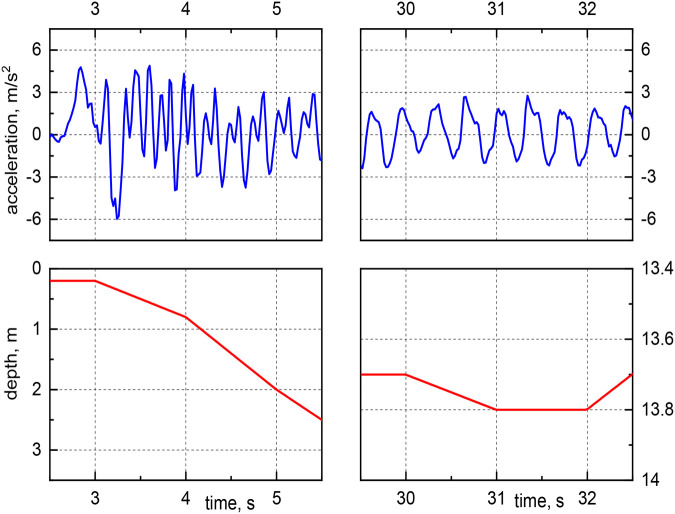
**Relative acceleration and current depth as a function of time for the same dive as in Fig. 3, for chosen time intervals, at the start of the dive descent (left panels) and at the dive bottom (right panels).** Note the different scales on the axes.

From the acceleration data, we estimated the instantaneous dive (or pitch) angle
(1)

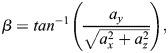
where *a*_*y*_ is the heaving acceleration in m/*s*^2^, *a*_*x*_ surging acceleration in m/*s*^2^ and *a*_*z*_ is, therefore, orthogonal accelerations in m/*s*^2^, on the plane perpendicular to *a*_*y*_ and *a*_*x*_. The relative acceleration, *a* in m/*s*^2^ plotted in Figs 3 and 4 is defined as

(2)

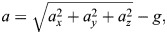


where g=9.81 m/*s*^2^ indicates the acceleration due to gravity.

**Fig. 5.**
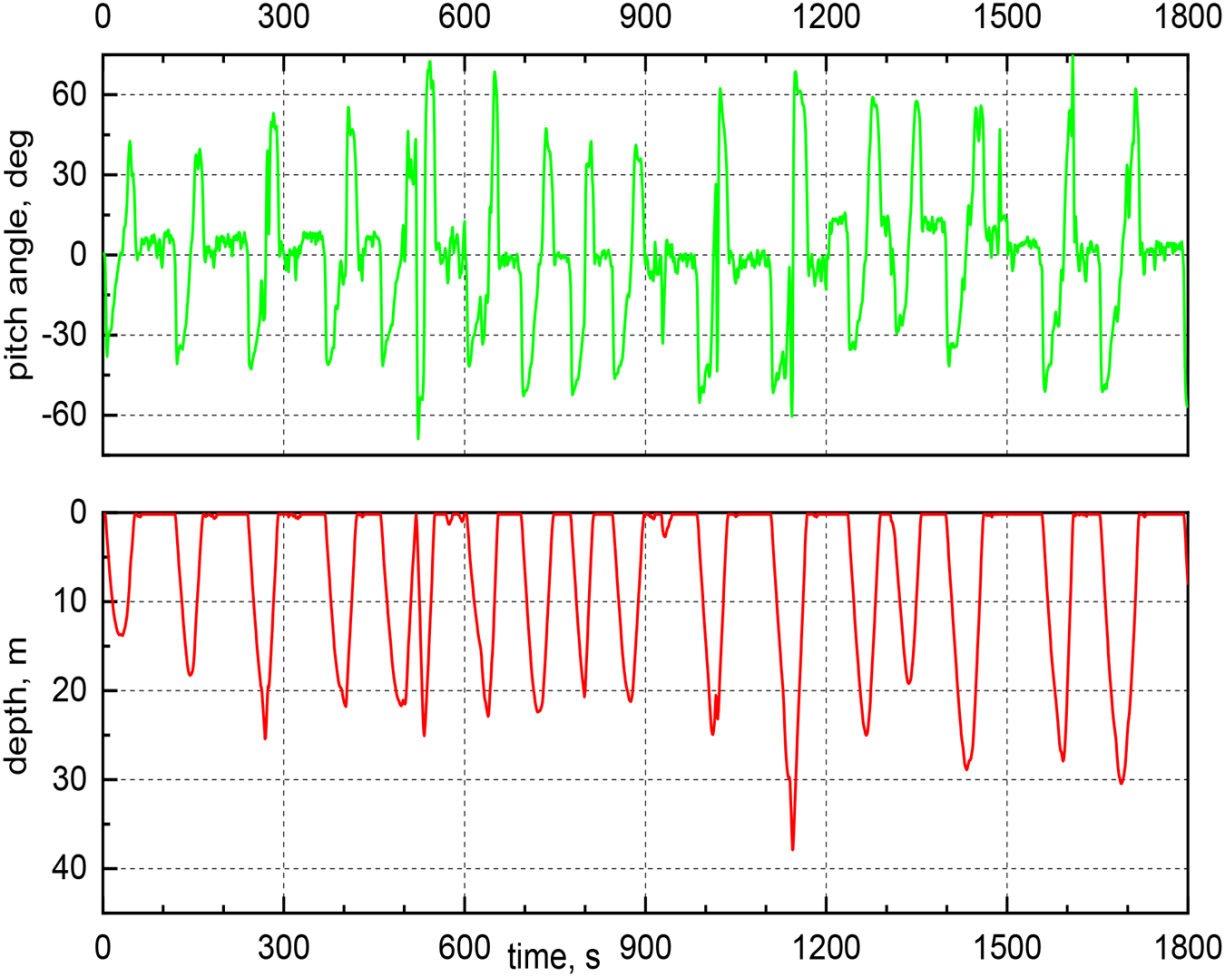
Dive (or pitch) angle and current depth as a function of time for some exemplary dives (the first dive is as in Fig. 3).

**Fig. 6.**
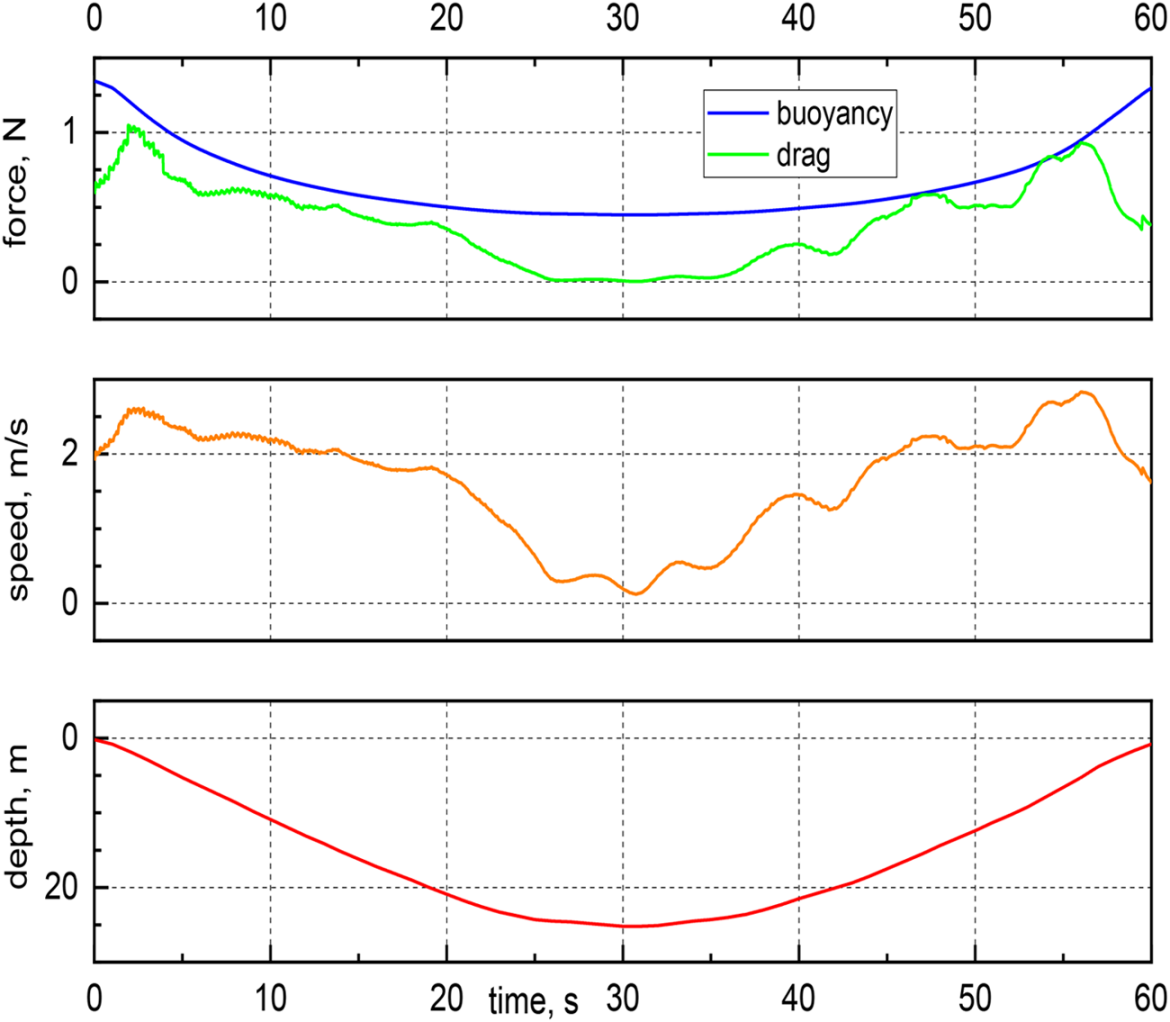
Buoyancy force, drag force, swimming speed and current depth as a function of time for a typical dive, not shown in other Figures above.

**Fig. 7.**
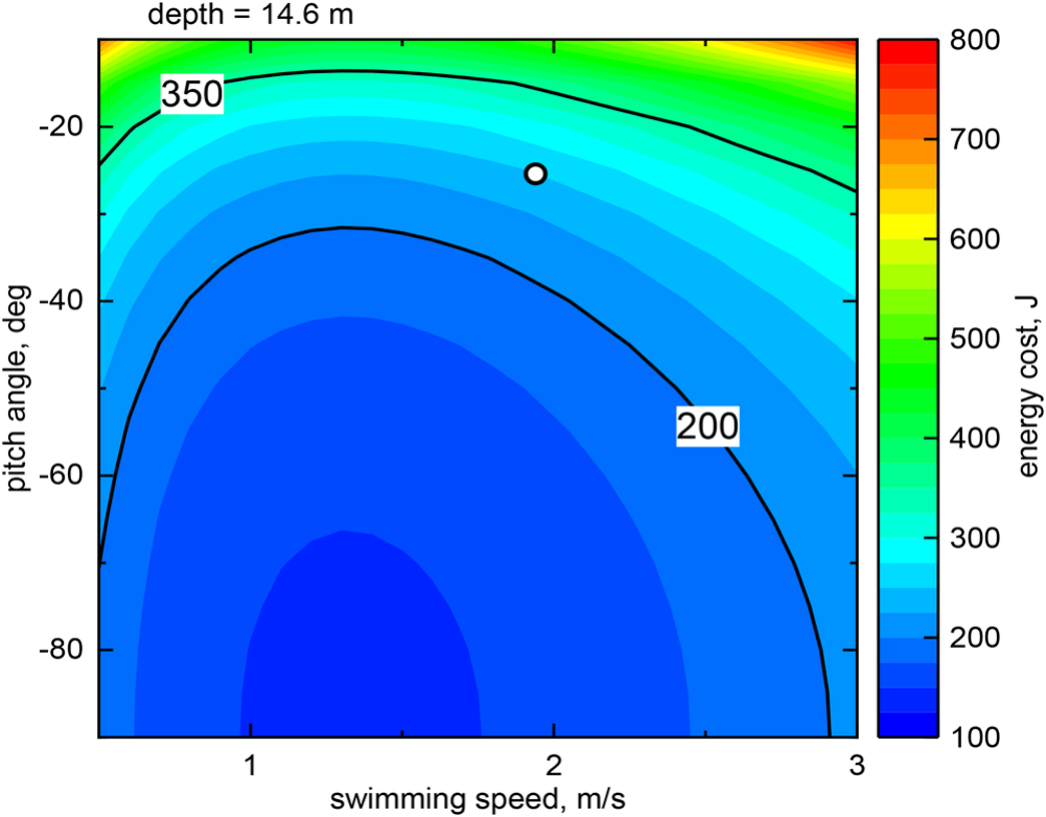
**Map of the energy cost *E*_*C*_ associated to the dive descent, plotted as a function of swimming speed during descent *U*_*d*_ and pitch angle during descent *β*_*d*_, at a given depth.** The white point indicates the energy cost computed using the actually obtained average values of depth, speed and pitch angle for all dives performed by free-ranging little penguin, as reported in Tables 1 and 2.

From the pitch angle, we calculated the swimming speed U in m/s, following the method mentioned in Watanuki et al. (2003), i.e.
(3)

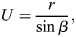
where r is the depth change rate, computed numerically from the depth data. Although depth was measured in 1 Hz frequency, in order to use depth data in the equation for estimating swimming speed, the values of depth were numerically interpolated to 50 Hz by using MATLAB R2019a.

The swimming speed U is then used to estimate the drag force *F*_*D*_ acting on the penguin, following Sato et al. (2010), Eqn 2.9; that is, we used the formula
(4)

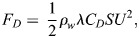
where *ρ*_*w*_=1027 kg/m^3^ is the density of seawater, and *λ*=0.576 indicates the ratio of the drag force acting on an active swimmer to that acting on a passive object [the numerical values reported in this section are the same used by Sato et al. (2010), if not stated otherwise]. The drag coefficient *C*_*D*_ is in general a function of the swimming speed *U*, as discussed, for example, by Lovvorn et al. (2001). Specifically, we set here *C_D_*=0.0051, using the formula given in Masud et al. (2022), with the estimated mean value of swimming speed *U* from the free-ranging dive data – see Table 1 – and with other parameters as in Masud et al. (2022). Additionally, we employ here the wetted surface *S*=0.0758 *m*^2^ of a frozen specimen of little penguin, reported by Lovvorn et al. (2001).

We estimate the buoyancy force *F*_*B*_ acting on the swimming penguin following Sato et al. (2010), Eqn 2.8, i.e., using the formula
(5)

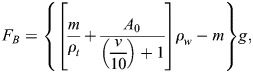
where *m*=1 *kg* indicates the bird mass. The tissue density *ρ*_*t*_=1020 *kg*/*m*^3^ is taken from Sato et al. (2010) and *A*_0_=0.000133 *m*^3^ indicates the air volume in the bird body, at sea surface and atmospheric pressure; this value was obtained considering that, as reported in Sato et al. (2010), Fig. 2 caption, the mass of an emperor penguin (*Aptenodytes forsteri*) can be set to 30 kg and the corresponding *A*_0_ to 0.004 *m*^3^, while, as just noted, the little penguin mass is here set to 1 kg. Last, in Eqn 5, *v* denotes the current depth, which was directly recorded using data loggers.

The required energy *E*_*V*_ to dive at a depth V is a function of the dive angle, buoyancy, dive depth, and drag force. Penguin's buoyancy at a given depth is generally considered as the weight of the seawater displaced by the penguin, minus its body weight. The seawater displacement is calculated by the air volume in the body 
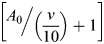
 and volume of animal tissue (m/*ρ*_*t*_) (Wilson et al., 1992; Sato et al., 2010). For the precise estimation of buoyancy force and energy cost during descent, actual measurement of air volume in the body is required, but is not available in the literature. Therefore, by using backtrack calculation from the reported data for emperor penguin (Sato et al., 2010), we have calculated the air volume in the body of little penguin. We have done it because air volume in the body has a proportional relationship with the body mass (Ponganis et al., 2015). By using the backtrack calculation, the initial air volume kept in the respiratory system and trapped in feathers at sea surface was found 0.000133 *m*^3^, which was used for subsequent calculations. Eqn 6. is derived to measure the required energy *E*_*V*_.
(6)

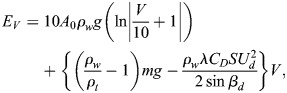
To compute the total energy necessary for diving to a depth of V, the assumption is made that there is no induced drag. However, it should be noted that swimming speed plays a crucial role in determining the mechanical energy cost as it has a direct relationship with speed (*E*_*V*_∝*U*^2^). In other words, the faster the swimming speed, the more energy is required to sustain the dive. However, it is worth noting that Eqn 6, which is used to calculate energy expenditure, is only applicable for vertical transit. This means that additional factors must be considered in the case of diving. For the purpose of accurately calculating the energy required during diving, it is important to take into account basal metabolic energy (BME) *M*_*V*_ (product of time and metabolic rate). At the time of descent, by using Eqns 7 and 8, we can determine the Basal metabolic energy (*M*_*V*_) expressed in J and Basal metabolic rate (BMR) *k* expressed in W, which is taken from Sato et al. (2010). By factoring in these additional variables, we can gain a more accurate understanding of the energy required for diving and better plan for these activities (McKechnie et al., 2006).
(7)

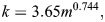
 and
(8)

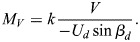
The calculation of total energy expenditure during descent involves using two types of energy: basal metabolic energy *M*_*V*_ and mechanical energy *E*_*V*_. The mathematical equation formulated for this specific purpose takes into account both forms of energy. By incorporating two key factors, propelling efficiency (ε_*p*_) and aerobic efficiency (ε_*A*_), Eqn 6 can be enhanced to increase the efficiency of the mathematical model (Hind and Gurney, 1997). Propelling efficiency refers to the ability of muscular movements to translate into forward thrust, while aerobic efficiency refers to the ability of chemical energy to be converted into muscular work. By considering both of these factors, Eqn 9 seeks to optimize the overall effectiveness of the model by introducing the total energy cost *E*_*C*_.
(9)

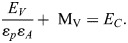
Hence, the energy cost ***E***_***C***_ associated to the dive descent, expressed in J, is here estimated following Sato et al. (2010), Eqn 2.13, i.e., using the formula
(10)

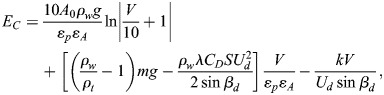
where ε_*p*_=0.85 and ε_*A*_=0.17, V indicates the depth reached by the penguin at a dive, and *β*_*d*_ denotes the mean value of the pitch angle during descent, obtained from the acceleration data and *U*_*d*_ swimming speed during descent.

Eqn 10 broadly accounts for the energy expended by a diving bird by accounting for the interaction with the water gravity and physical effort in mechanical terms in addition to accounting for the physiological cost of the effort. It should also be noted that it accounts for the descent phase when the effort is made, penguins ascent to the surface with a minimal effort due to buoyancy and no flapping action. The first term accounts for the effort to overcome buoyancy taking into consideration physiological efficiency (i.e. the required energy is higher than the physical value). We would like to draw the reader's attention to the ratio V/10 that reflects the fact the hydrostatic pressure increases about one atmosphere for every 10 m of water depth; as the little penguin is not a highly proficient diver, the hydrostatic pressure in our records would go up to about six times the atmospheric one meaning that the pressure will not significantly affect the rib cage shape. The second term accounts for the positive contribution of the gravity as penguins are not naturally buoyant, and the negative drag force. Regarding the drag estimate, it is a quasi-steady estimate, assuming that the drag coefficient could be averaged as the projected wet area remains constant during flapping; furthermore, as propulsion counteracts the natural lift and suppresses the lift-induced drag, such an assumption is quite reasonable to make. Regarding the drag experienced by the flapping wings, it is converted into propulsive force and not encountered directly as a resisting force. The third term accounts for the shape of the dive as it incorporates the diving angle, showing that minimal energy is expended when diving vertically when sine is 1. In summary, the model as seen is limited to mechano-physiological contribution and completely ignores the thermodynamic part of the energy needed to maintain body temperature in the cold waters for example. It simply reflects the mechanical work under the physiological constraints.

Finally, the *CoT* associated to the dive descent is set equal to the energy cost per unit of mass and depth, i.e.
(11)

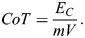
The non-dimensional indicators of propulsive performance employed in the study are defined following Masud et al. (2022). The Strouhal number (*St*) is written as
(12)

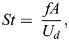
where *f* denotes the wingbeat frequency, *A* indicates the corresponding amplitude, and *U*_*d*_ is the animal swimming speed ‒ note that in Masud et al. (2022) the propulsive amplitude was set to the bird wingspan, equal to 0.12 m for little penguins. Kato et al. (2006) reported that little penguins usually do not change the amplitude of strokes during the descent phase; therefore, we believe that our hypothesis of taking the wing length as a wingbeat amplitude will not affect the result significantly (Kato et al., 2006). Moreover, note be mentioned that we estimated the wingbeat frequency from the heaving acceleration data by using Fourier transformation, and this was done only for the descent period after the penguin reached at least 1 m depth following the methodology mentioned by Kikuchi et al. (2015). The wingbeat frequency was analysed using the heaving acceleration signal because they are the most sensitive to undulation in the birds’ body resulting from flipper beats (Zimmer et al., 2011; Kikuchi et al., 2015).

Finally, we have estimated the Reynolds number (*Re*), which is written as
(13)

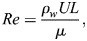
where L indicates a characteristic scale of the studied flow, set to the bird height in Masud et al. (2022), equal to 0.36 m for little penguins, and μ is the dynamic viscosity of water, set to 0.00141 kg/m/s in Masud et al. (2022).
